# Intravenous infusion of nicotinamide adenine dinucleotide (NAD^+^) versus nicotinamide riboside (NR): a retrospective tolerability pilot study in a real-world setting

**DOI:** 10.3389/fragi.2026.1652582

**Published:** 2026-02-02

**Authors:** Kirsten Reyna, Greer Heinzen, Nikita Patel, Marie Ritter, Alexandra Siojo, Henry Legere, Rachele Pojednic

**Affiliations:** 1 RestoreLabs, Restore Hyperwellness, Austin, TX, United States; 2 University of Connecticut, Storrs, CT, United States; 3 Nutrition and Health Innovation Research Institute, School of Medical and Health Sciences, Edith Cowan University, Joondalup, WA, Australia; 4 Stanford Lifestyle Medicine, Stanford Prevention Research Center, Stanford University School of Medicine, Palo Alto, CA, United States

**Keywords:** biomarkers, Intravenous, NAD^+^, nicotinamide adenine Dinucleotide, nicotinamide Riboside, safety

## Abstract

**Background:**

Nicotinamide adenine dinucleotide (NAD^+^) is a cofactor for NAD^+^-dependent enzymes that regulate DNA repair, cellular metabolism, and immune function. Supplementation with NAD^+^ and its precursors is commonly used to prevent age-related disease and extend healthspan. Commercial clinical and wellness settings increasingly provide intravenous (IV) NAD^+^ and nicotinamide riboside (NR) despite limited evaluation of safety and effectiveness. These considerations are important, as NAD^+^ and its precursors differ in reported side effects, cellular uptake, and metabolism. This study sought to compare commercially administered NAD^+^ IV and NR IV in humans by evaluating infusion time, tolerability, safety markers, and metabolic outcomes.

**Methods:**

A retrospective review of electronic medical records was conducted in clients from a commercial setting. Participants received four consecutive days of 500 mg NAD^+^ IV or NR IV, with 30 days follow-up. Primary outcomes included reported symptoms, total infusion time, blood pressure, resting heart rate, and biomarker assessments (ALT, AST, hsCRP, BUN/creatinine, and TSH). Exploratory analyses included metabolic biomarkers (HbA1c, fasting glucose, HDL-C, LDL-C, and triglycerides).

**Results:**

Tolerability differed between groups. Participants that received NAD^+^ IV reported moderate to severe gastrointestinal symptoms, increased heart rate, and chest pressure during infusions. Participants receiving NR IV experienced minor tongue, jaw, and arm tingling and mild cramping during infusion. All symptoms resolved upon infusion completion. Moderate to severe symptoms with NAD^+^ IV resulted in longer infusion times compared to NR IV, averaging 97 min versus 37 min, respectively. No significant changes were observed in ALT, AST, hsCRP, BUN/creatinine, or TSH. Alkaline phosphatase (ALP) decreased significantly in the NAD^+^ IV group only, with values remaining within normal reference ranges. The NR IV group demonstrated a significant reduction in HbA1c, whereas the NAD^+^ IV group showed a significant reduction in HDL-C. Neither group exhibited changes in fasting glucose or LDL-C over the 30-day period.

**Conclusion:**

This study directly compared commercially administered NAD^+^ IV and NR IV, providing preliminary real-world evidence of infusion tolerability and short-term safety. Exploratory metabolic outcomes were variable and warrant further investigation. Additional studies are needed to evaluate dosage and effectiveness beyond 30 days.

## 1 Introduction

Nicotinamide adenine dinucleotide (NAD^+^) is an essential pyridine nucleotide, required for energy (ATP) production in the mitochondria via redox reaction (Covarrubias et al., 2021). NAD^+^ is also a cofactor in NAD^+^ dependent enzymes that regulate DNA repair, gene expression, cellular metabolism, and immune function (Amjad et al., 2021). The dysregulation of NAD^+^ homeostasis has been associated with metabolic (Wu et al., 2016; Kane and Sinclair, 2018) and neurodegenerative (Lautrup et al., 2019) disorders across the lifespan. Moreover, tissue and cellular NAD^+^ have been shown to decline with age (Covarrubias et al., 2021; Chini et al., 2024) leading to reduced physiologic function and contributing to age-related diseases including chronic inflammation, cognitive decline, metabolic disease, sarcopenia and even some cancers (Covarrubias et al., 2021). NAD^+^ repletion has emerged as a potential therapeutic strategy to mitigate these related disorders and chronic diseases of aging.

NAD^+^ itself is present in the extracellular fluid in very small concentrations of 0.1 and 0.5 μM (Adriouch et al., 2012; Gasparrini et al., 2021) and the concentration in plasma has been found to be approximately 500 times lower than in peripheral blood mononuclear cells (Saqr et al., 2023). There are concerns that extracellular NAD^+^ is considered a danger signal released during cell damage to alert the immune system (Adriouch et al., 2012) and can have proinflammatory effects (Gasparrini et al., 2021). Therefore at high concentrations, NAD^+^ is hydrolyzed into NMN and converted by CD73 into NR or through CD38 and CD157 into nicotinamide (NAM) which are then directly absorbed into the cell through molecule specific transporters (Covarrubias et al., 2021; Sauve et al., 2023; Grozio et al., 2019; Damgaard et al., 2022; Kropotov et al., 2021). This ability to cross cell membranes makes NR and NMN potentially more efficient precursors to increase intracellular NAD^+^ levels than administering NAD^+^ directly.

Supplementation with NAD^+^ precursors has shown promise in attenuating NAD^+^ declines (Martens et al., 2018) and cellular dysfunction (Elhassan et al., 2019; Campbell, 2022; Zhong et al., 2022; Sohouli et al., 2024). Historically examined methods of NAD^+^ supplementation in humans include ingestion of commercially available oral precursors (i.e., nicotinamide riboside (NR) and nicotinamide mononucleotide (NMN)) (Freeberg et al., 2023). More recent studies have investigated intravenous (IV) NR and NAD^+^ applications in both preclinical (Damgaard et al., 2022) and human (Grant et al., 2019) trials, demonstrating increases in tissue and plasma NAD^+^ concentrations, respectively. However, there are no trials to date that have compared physiologic or clinical outcomes directly between precursors, nor in methods of application. This is a critical gap in the literature as NAD^+^ and its precursors, NR and NMN, differ in metabolism and cellular uptake (Amjad et al., 2021).

With regard to oral supplementation, NAD^+^ can be synthesized *de novo* from L-tryptophan through the kynurenine pathway or from vitamin precursors like niacin (B3) nicotinic acid (NA), via the Preiss–Handler pathway, or through oral NAM, NR or NMN via the salvage pathway (Covarrubias et al., 2021). NAD^+^ itself is unable to undergo direct intestinal absorption or cellular uptake (Nikiforov et al., 2011). However, diet-derived NAD^+^ precursors, as well as orally administered NR and NMN, undergo complex intestinal absorption mediated by gastric microbiome interference, and substantial first-pass metabolism, resulting in variable conversion to downstream metabolites before reaching the systemic circulation (Zhang et al., 2025; Canto, 2022; Lautrup et al., 2019). Nevertheless, a portion of these metabolites, and, in some cases, intact precursors, enter the bloodstream and contribute to cellular NAD^+^ synthesis via tissue-specific transport and enzymatic pathways (Zhang et al., 2025; Sauve et al., 2023; Gross and Henderson, 1983; Nikiforov et al., 2011).

Orally delivered NR and NMN are converted into bioavailable NAD^+^ in mammalian (Ratajczak et al., 2016) and human cells (Covarrubias et al., 2021; Airhart et al., 2017; Chini et al., 2024), peaking between three and 8 hours after intake (Trammell et al., 2016; Airhart et al., 2017). Significant increases of whole blood NAD^+^ can be achieved after 1 week of daily ingestion of oral NR at 300 mg or 1,000 mg doses (Conze et al., 2019) as well as doses of 2000 mg (Airhart et al., 2017). Oral NR supplementation of 1000 mg/day has also been shown to elevate levels of NAD^+^ in human peripheral blood mononuclear cells by ∼60% after 6 weeks (Martens et al., 2018). Even higher oral doses of 3000 mg per day have also been demonstrated to be safe and resulted in a 5-fold increase in blood NAD^+^ levels after 10-week (Berven et al., 2023).

Despite the demonstrated tolerability, safety, and increases in NAD^+^ concentrations observed with oral precursor supplementation, intravenous NAD^+^ and, more recently, intravenous NR therapies have become widely available in commercial clinics, likely having roots in early use for addiction disorders (O’hollaren 1961). However, human data evaluating the efficacy and health effects of intravenous administration remain limited. It is plausible that IV administration may provide similar, or even improved, NAD^+^ support due to the increase in bioavailability. There may also be clinical implications as deficiencies or suboptimal levels of the dietary NAD^+^ precursors, niacin and tryptophan, can also occur in chronic alcohol use disorder (Yogi et al., 2024) and in patients with inflammatory bowel disease (Harries and Heatley, 1983) or Hartnup disease (Kleta et al., 2004). In these populations, intravenous therapy may represent a potential alternative route of administration for individuals with gastrointestinal disorders or those who do not tolerate oral supplementation.

While intravenous administration may be considered for both healthy and clinical populations, important questions remain regarding the safety, tolerability, and effectiveness of NAD^+^ IV and NR IV. Specifically with NAD^+^, common clinician and patient reported side effects include nausea, malaise, diaphoresis, stomach cramping, headache which may be directly related to the proinflammatory environment created by increasing extracellular NAD^+^ to supraphysiologic concentrations (Adriouch et al., 2012; Gasparrini et al., 2021). Anecdotal reports indicate that these side effects necessitate slow, prolonged infusions to minimize discomfort. NR has yet to be studied in its intravenous form in humans, specifically in comparison to NAD^+^. The absence of comparative research between NAD^+^ IVand NR IV creates significant gaps in understanding their safety, tolerability and metabolic impacts. To address this critical gap in the literature, this study sought to directly compare commercially available intravenous NAD^+^ and NR administration in humans by evaluating infusion time, tolerability, safety markers, and metabolic outcomes.

## 2 Materials and methods

### 2.1 Study design

A retrospective review was performed on the electronic medical records (AdvancedMD, South Jordan, UT) of clients in a commercial setting (Restore Hyperwellness, Austin, TX) during and 30 days after infusion of commercial NAD^+^ IV (Empower Pharmacy, Houston, TX) or NR (Niagen Biosciences, Wells Pharmacy, Houston, TX) products. Records were included if the client was over the age of 18, not pregnant, and did not have a family or recent history of cancer, which were all contraindications of IV therapy at the commercial clinic. Clients completed a commercial informed consent for infusions received at the studio, including their consent for deidentified data to be used for research purposes. The BRANY institutional review board approved the study as exempt and waived the need for additional informed consent owing to its minimal risk nature (#25–12–096–1,589).

One to 4 days prior to infusion, clients were cleared for IV infusion by a nurse practitioner and had baseline blood drawn. Clients were asked to complete an overnight fasting protocol, which consisted of not eating or drinking 8 h prior to their blood draw appointment at the studio for baseline biomarker assessment. Clients began their first IV infusion studio visit with blood pressure and body composition measurements, and completed surveys on quality of life, physical activity, and dietary behavior. Clients then underwent IV placement and infusion. Nurses monitored clients during the infusion periods while recording infusion time and symptoms. At 30 days post-treatment, body composition and fasting blood tests were repeated to assess the durability of biometric and biomarker responses beyond the acute post-infusion period. Clients also shared personal biometric data for 30 days post infusion monitored remotely by a commercially available photoplethysmography device.

### 2.2 Commercial IV infusion protocol

Clients were asked to repeat the overnight fasting protocol, prior to their in-person appointment and to also avoid exercise 24 h before attending their infusion visits. Nurses reconstituted 500 mg of lyophilized NAD^+^ or NR powder with bacteriostatic water, as directed by supplier, and diluted it in 500 mL of normal saline. Clients were intravenously infused with a 22-gauge IV catheter needle. Identical infusions were repeated for four continuous days at the same time each day, which is the typical commercial “loading dose” recommended by the compounding pharmacy (Lyo, 2026). Time of infusion start, and stop were recorded as well as subjective experiences during IV administration. Clients controlled their own infusion rate based on tolerance.

### 2.3 Blood biomarkers

Blood biomarkers at baseline and 30-day post infusion included serum hsCRP, measures of liver function (ALT, AST), renal function (eGFR, BUN/Creatinine ratio, electrolytes), thyroid (TSH), and metabolic analytes (fasting glucose, HbA1c, and serum lipids, including total cholesterol, LDL HDL, and triglycerides). All blood was drawn from the median cubital vein with a 22-gauge IV catheter needle. Drawn samples were sent to Quest Diagnostics (Secaucus, NJ, USA) for analysis. High-Sensitivity C-Reactive Protein (hsCRP) was analyzed with an immunoturbidimetric assay. Fasting glucose, serum lipids (HDL and LDL), triglycerides, creatinine, urea nitrogen (BUN) and liver enzymes were analyzed with spectrophotometry. During the 4 days of infusion whole blood NAD^+^ concentrations were sampled via dried blood spot (DBS). Finger punctures were conducted using a lancet. Blood was then applied to the circles on the DBS card and allowed to dry for a minimum of 3 h. Dried cards were stored at −20 °C until shipment for analysis by LC/MS via a commercial lab (Revvity Inc, Waltham, MA).

### 2.4 Body composition

Body composition was measured using InBody multi-frequency bioelectrical impedance machines (Inbody570, Inbody USA, Cerritos, CA) which has been validated to provide acceptable body composition results compared to dual-energy x-ray absorptiometry (DXA) (McLester et al., 2020). Clients were not assessed for hydration status prior to body composition testing. There were three overarching categories measured with InBody: fat mass, lean muscle mass, and water content, with individual data points including weight, body mass index (BMI), body fat mass (BFM), percent body fat, visceral fat level, arm circumference, and BFM and BFM% for left/right arms, left/right legs, and trunk. Height was provided by the clients and was not measured directly.

### 2.5 Photoplethysmography

Photoplethysmography ring devices (OURA, Oulu, Finland) were fitted to the client’s finger of choice and continuously tracked biometrics throughout the 4-day loading period and 30-day post-treatment, including resting heart rate, heart rate variability, respiration, sleep, physical activity, stress levels, and body temperature. OURA monitors: wear time, nocturnal heart rate, respiration (30-s resolutions), and heart rate variability (HRV) were indirectly with an infrared sensor that detects a client’s arterial pulse on the finger using photoplethysmography at 250 Hz, negative temperature coefficient (NTC) sensor for body temperature and 3D accelerometry for movement. To sync continuously, clients downloaded the OURA web application and had access to their data at all times. OURA outputs were calculated using body metrics (sex, age, body mass, and height) entered by the client. Researchers monitored and downloaded all data through a remote dashboard that was updated at noon daily.

### 2.6 Surveys

Survey data was collected at baseline and 30-day post-loading using validated assessments. clients completed the following PROMIS surveys-quality of life (PROMIS Global Health Scale v1.25) (Hays et al., 2009), fatigue (Neuro-QOL Item Bank v1.0 –Fatigue–Short Form) (Lai et al., 2011), pain intensity (PROMIS Scale v2.0 – Pain Intensity 3a) (PROMIS Pain Intensity, 2025), cognitive function (PROMIS Item Bank v2.0 – Cognitive Function - Abilities Subset—Short Form 4a) (Lai et al., 2014) and sexual health (PROMIS Scale v2.0 - Sexual Function and Satisfaction: Interest in Sexual Activity) (Weinfurt et al., 2015), and sleep (PROMIS® Short Form v1.0 - Sleep-Related Impairment - Short Form 4a) (Yu et al., 2011). Additionally, clients completed an International Physical Activity Questionnaire (IPAQ Short Form) (Lee et al., 2011) and PRIME dietary screen (PRIME Screen) (Rifas-Shiman et al., 2001).

### 2.7 Statistical analysis

There were two primary analyses. First, the comparison of infusion time and client experiences during the 4-day loading period; and second, baseline and post-treatment comparison of measured metabolic biomarkers, blood pressure, and resting heart rate within and between each treatment group.

Data were checked for normality using Shapiro–Wilk test and normality distribution plots. Data examining infusion time, that did not violate assumptions of normal distribution were analyzed using t-tests, and data with violations of normal distribution were analyzed using Wilcoxon rank-sum tests (also known as Mann–Whitney U test). Mixed-design ANOVA (within-subject factor = baseline/post-treatment, and between-subject factor = Niagen product, NAD^+^ or NR) were used to analyze metabolic biomarkers, physical activity, diet and body composition. Data were also checked for equal variances using Levene’s Tests, if this assumption was violated, data were analyzed using Welch’s Test. All data cleaning, analysis, and statistical modeling was done in R using tidyverse (Wickham et al., 2019) and rstatix (Kassambara, 2023) packages.

## 3 Results

Six (n = 6) clients received 500 mg of intravenous NAD^+^ and eight (n = 8) clients received 500 mg intravenous NR. Baseline characteristics are presented in Table 1.

**TABLE 1:** Baseline characteristics, including biological sex, age, height, weight, blood pressure, and resting heart rate for NAD+ and NR groups. Values presented as mean ± standard deviation.

Parameters	NAD^+^ (n = 6)	NR (n = 8)
Gender *(n)*		
Female	2	4
Male	4	4
Age	29.4 (3.17)	37.125 (7.94)
Average height (inches)	66.6 (6.11)	67.71 (4.64)
Average weight (pounds)	201.14 (92.72)	181.56 (55.75)
Systolic blood pressure	131.48 (15.76)	120.35 (15.75)
Diastolic blood pressure	82.67 (12.94)	79.39 (10.53)
Resting heart rate	72.66 (4.48)	64.27 (2.55)

### 3.1 Adverse events and adverse experience

There were no documented adverse events although one client in the NR group experienced an adverse experience (i.e., severe cramping coinciding with menstruation) during the second day of infusion that led to a temporary discontinuation of infusion. The experience was considered mild and the client later continued with their third and fourth infusion with no additional adverse experience. All clients (n = 6) in the NAD^+^ group reported moderate to severe abdominal cramping, diarrhea, nausea, vomiting, increased heart rates, pain in throat, congestion, and chest pressure during infusion. These adverse experiences ceased immediately upon infusion completion. Some, but not all, clients (n = 5) in the NR group experienced minor tongue, jaw and arm tingling and minor cramping during infusion. Similar adverse experiences have been reported during NAD^+^ and NR infusion study currently in preprint and were not unique to this administration (Hawkins et al., 2024).

### 3.2 IV infusion time

**FIGURE 1:**
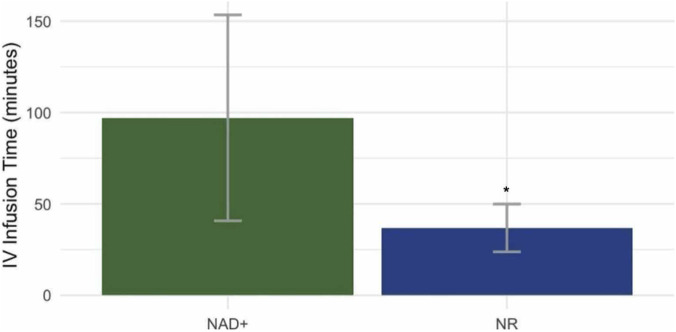
Infusion time: Clients received four IV infusions of either Nicotinamide Adenine Dinucleotide (NAD^+^) or Nicotinamide Riboside (NR) 500 mg in 500 mg of saline over a 4 day loading period. Each client was allowed to control their own flow rate as tolerated. The NAD^+^ group had an average IV infusion time of 96 min, while the NR group had an average IV infusion time of 37 min (p < 0.05*). The NR group average IV infusion times was 60% lower than the NAD^+^group. Barplot shows mean values ±standard deviation.

Clients that received NAD^+^ had a 4-day average total infusion time of 97 ± 56.33 min. Those that received NR had a 4-day average infusion time of 37 ± 13.08, resulting in a 60% reduction compared to the NAD^+^ clients (Figure 1).

### 3.3 Safety

The primary analysis focused on examining liver enzyme serum levels to evaluate the safety of NAD^+^ and NR IV administration. Liver enzyme tests included alkaline phosphatase (ALP), alanine aminotransferase (ALT), and aspartate aminotransferase (AST). ALT and AST had no statistically significant change between baseline and post-treatment levels (p > 0.05). However, ALP was reduced significantly in the NAD^+^ group after 30 days but not in the NR group, resulting in significant differences between NAD^+^ and NR (Table 2). There were no statistically significant changes in hsCRP, BUN/Creatinine, or TSH from baseline to 30-day post-treatment in either group (Supplementary 1). Quest Diagnostics was unable to provide a calculated eGFR as client age or sex information was reported as not available at the time of analysis. There was no significant change in systolic or diastolic blood pressure for either group during the 4 days of infusion (Table 3).

**TABLE 2:** The mean change for NAD+ and NR clients from baseline to 30 days post-treatment. Positive values indicate clients had a mean increase from baseline to post-treatment, and negative values indicate clients had a mean decrease from baseline to post-treatment. Values presented as mean change ± standard deviation. Corresponding p-values were reported when statistical significance was found.

Biomarkers	Treatment Group		P-Value	
	NAD^+^ (n = 6)	NR (n = 8)	Pre/Post comparison	NAD+/NR comparison
ALT (U/L)	8.67 (15.01)	0.57 (7.23)	​	​
AST (U/L)	9.00 (13.86)	−2.00 (3.32)	​	​
ALP (U/L)	−7.00* (2.65)	3.86 (7.01)	p = 0.04*	p < 0.01
LDLc (mg/dL)	9.80 (35.83)	0.88 (14.76)	​	​
HDLc (mg/dL)	−5.80* (3.42)	0.13 (5.17)	p = 0.02*	p = 0.03
Triglycerides	−17.20 (21.59)	−9.75 (24.96)	​	​
HbA1c	0.02 (0.11)	−0.13* (0.10)	p = 0.01*	p = 0.04
Glucose	6.67 (19.43)	1.29 (9.55)	​	​
TSH	0.06 (0.94)	0.02 (0.88)	​	​
BUN	−0.33 (1.15)	1.57 (3.55)	​	​
Creatinine	−0.03 (0.09)	0.04 (0.12)	​	​
hsCRP	0.62 (0.74)	0.53 (2.63)	​	​
Resting heart rate	−3.60	−1.22	​	​

**TABLE 3:** Average blood pressure (mm Hg) for each infusion day by product type. Typically reported as Systolic/Diastolic, with the normal blood pressure being less than 120/80. Values reported as mean ± standard deviation. Systolic = SBP, Diastolic = DBP.

Infusion day	SBP	DBP
NAD^+^ (n = 6)		
Day1	134.33 (13.28)	81.33 (13.84)
Day2	132.83 (17.01)	84.33 (13.88)
Day3	129.25 (20.42)	84.75 (12.09)
Day4	128.20 (17.73)	80.60 (15.21)
NR (n = 8)		
Day1	121.88 (18.83)	82.38 (8.91)
Day2	119.29 (16.73)	79.14 (12.06)
Day3	118.63 (15.09)	74.88 (12.62)
Day4	121.50 (15.24)	81.13 (8.64)

### 3.4 Metabolic biomarkers, physical activity, diet and body composition

**FIGURE 2:**
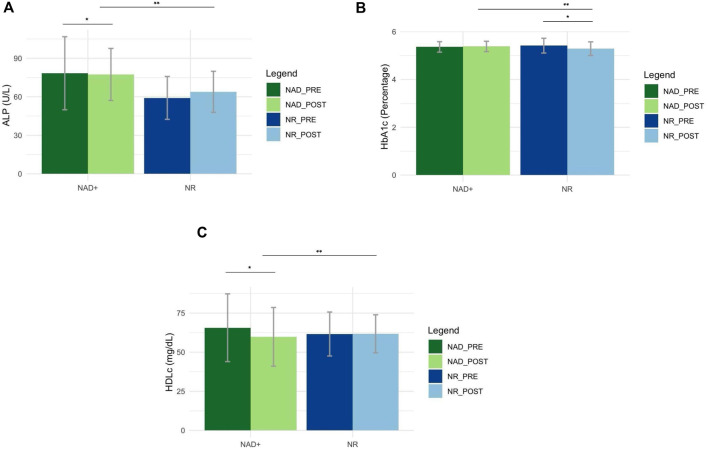
Metabolic Biomarkers. **(a)** Alkaline Phosphatase (ALP): Among the liver enzyme tests, statistically significant differences were found in ALP values across groups (p = 0.04), and between baseline and post-treatment in the NAD^+^ group only (p < 0.01). The NAD^+^ group saw a significant increase in ALP, while the NR group saw a non-significant increase in ALP. Average ALP values were within normal limits at all collection timepoints. Normal reference ranges for Males: 36 - 130 U/L, and Females: 31 - 125 U/L. Bar plot values represent mean and include error bars. **(b)** Hemoglobin A1c (HbA1c): The NR group had a significant decrease in HbA1c (p =0.01*), while the NAD^+^ group had a negligible increase. The significant difference in the NR group contributed to a statistically significant difference in HbA1c across NAD^+^ and NR groups (p = 0.04). Despite significant differences within and across groups, mean HbA1c values were within normal reference range, <5/7%. Bar plot values represent mean and include error bars. **(c)** High Density Lipoprotein (HDLc): The NAD^+^ group saw a statistically significant decrease in mean HDLc values (p = 0.02*) after 30 days, while the NR group saw a non-statistically significant increase in mean HDLc values (p > 0.05) after 30 days. The significant difference in the NAD^+^ group contributed to a significant difference in mean HDLc values across NAD^+^ and NR groups (p = 0.03**). Despite significant differences within and across groups, mean HDLc values were within normal reference ranges, Males ≥ 40 mg/dL and Female ≥ 50 mg/dL. Bar plot values represent mean and include error bars.

Mixed-design ANOVA revealed that the NR group demonstrated a significant reduction in HbA1c levels (p < 0.05) after 30 days (Figure 2). The NAD^+^ group experienced a significant reduction in HDLc values (p < 0.05) with a non-significant rise in LDLc after 30 days. Between NAD^+^ and NR groups, there were no statistically significant changes in fasting glucose or LDLc from baseline to 30-day post-treatment. Supplementary 1 contains averages at baseline and 30-day post treatment.

Select surveys were analyzed using Wilcoxon rank-sum tests to examine differences at within-subject and between-subject levels. IPAQ showed no significant changes in physical activity from baseline to 30-day post treatment or between NAD^+^ and NR groups. PRIME Screen recorded no significant changes in dietary behavior (including multivitamin supplementation) and InBody showed no significant changes in body composition from baseline to 30-day post treatment or between groups. Participants in both groups reported little to no changes in alcohol consumption from baseline to 30-day post treatment. Changes in metabolic biomarkers occurred without significant differences in physical activity, dietary habits, or body composition from baseline to 30-day post-treatment.

## 4 Discussion

The increasing clinical and wellness use of commercially available intravenous NAD^+^ and NR has raised questions about tolerability, safety, and effectiveness. While NAD^+^ infusions have been associated with side effects likely driven by supraphysiologic extracellular NAD^+^ levels and their proinflammatory effects, there is limited evidence on how to optimize administration to minimize adverse effects. Anecdotal strategies such as using slow, prolonged infusions have been proposed, but formal studies are lacking. This study was designed to address these gaps by comparing intravenous NAD^+^ and intravenous NR administration in humans, with a focus on tolerability, infusion time, safety markers, and metabolic outcomes. The findings of this study demonstrate that NR had fewer adverse experiences compared to NAD^+^ infusions, demonstrated a 60% reduction in infusion time, and is safe to administer. Liver enzymes remained stable or were reduced, and hsCRP remained unchanged 30-day after infusion regardless of product. Secondary analysis of metabolic biomarkers indicated potential improvements in glucose metabolism 30-day after NR infusion, and mixed findings in lipid metabolism 30-day after NAD^+^ infusion.

### 4.1 Adverse experience and infusion time

Only three recent studies have examined intravenous administration of NAD^+^ and its precursor nicotinic acid in humans (Grant et al., 2019; Nelson et al., 2012; Gadegbeku et al., 2003). Two studies infused nicotinic acid to understand hemodynamic outcomes (Gadegbeku et al., 2003) and lipid metabolism (Nelson et al., 2012). One investigated NAD^+^ infusion directly (Grant et al., 2019) in order to describe NAD^+^ metabolism and cellular absorption kinetics. One study (in preprint) infused NR and NAD^+^ to compare NAD^+^ concentrations in whole blood along with safety and immune biomarkers (Hawkins et al., 2024).

One of the primary outcomes of the current study was to classify subjective experience during infusion as adverse experiences have been reported during NAD^+^ infusion including moderate to severe gastrointestinal symptoms, discomfort in throat, chest, and legs, and hot flashes (Niacin, 2025). In a study currently published in pre-print, Hawkins and colleagues (Hawkins et al., 2024) have demonstrated that NR IV results in fewer and milder side effects. Specifically, they demonstrated that 500 mg NR IV was well tolerated, with no significant adverse events and only minor, transient infusion-related experience. Participant experience from the current study also demonstrated reduced adverse experience with NR IV, with some participants, although not all, experiencing minor tongue, jaw and arm tingling and minor cramping during infusion. This is in contrast to NAD^+^ IV, with all participants reporting moderate to severe abdominal cramping, diarrhea, nausea, vomiting, increased heart rates, pain in throat, congestion, and chest pressure during infusion. These adverse experiences ceased immediately upon infusion completion, although did cause participants to reduce infusion rate in the NAD^+^ IV group.

Adverse experience is significant in the current IV application as it dictates client-controlled infusion time. Only one prior study has examined NAD^+^ infusion under a controlled 6-h administration but did not report subjective experience (Grant et al., 2019). Two recent studies administering nicotinic acid reported titrating infusion to 2.8 mg/min during 20 min to “minimize symptoms”, but symptoms were not outlined or monitored (Nelson et al., 2012; Gadegbeku et al., 2003). As participants were unable to change infusion rate, it is unclear whether adverse experience would have played a role in infusion time in any of these prior investigations. More recently, one preprint randomized controlled trial directly compared adverse experience and infusion time between NAD^+^ IV to NR IV and demonstrated reduced infusion time with NR IV(Hawkins et al., 2024). In that investigation by Hawkins and colleagues (Hawkins et al., 2024), the mean tolerable infusion time for NR IV was 75% less than that of NAD^+^ IV during one infusion. This is consistent with the average reduction of infusion time by 60% over four consecutive days of infusions in the current study. In both investigations, longer infusion times with NAD^+^ IV were due to adverse experiences during administration. In the time period after infusion, no adverse events were reported during and up to 14-day in a prior investigation (Hawkins et al., 2024), and no adverse events were reported during and up to 30-day after infusion in the current study.

### 4.2 Safety

When examining safety, NR IV and NAD^+^ IV both exceeded the recommended dietary allowance (RDA) of niacin (vitamin B3) which is set at 16 mg niacin equivalence (NE) for men and 14 mg NE for women, increasing to 18 mg NE for pregnant women and 17 mg during lactation (Niacin, 2025). However, oral supplementation of niacin, including NR, often exceeds the RDA with limited reported side effects. Numerous clinical studies have consistently demonstrated the safety of oral NR even at doses well above the RDA. Several studies have supplemented 1000 mg/day of NR with no clinically relevant adverse effects in healthy adults (Conze et al., 2019; Airhart et al., 2017), as well as clinically vulnerable populations with cognitive impairments (Wu et al., 2025) and chronic kidney disease (Ahmadi et al., 2023).

With regard to increases in whole blood and serum NAD^+^ during and after NAD^+^ IV and NR IV, there are preliminary data to demonstrate significant differences within and between groups. With regard to NAD^+^ concentrations itself, Grant and colleagues demonstrated a rise in plasma NAD^+^ 2 hours post infusion start with increased urinary NAD^+^ concentrations measured after 6 h (Grant et al., 2019). In an preprint study (Hawkins et al., 2024) NR IV led to significantly higher whole blood NAD^+^ levels, as measured by dried blood spot analyses, NR IV appeared to promote increases in peak NAD^+^ concentration by 20.7% relative to baseline, and acutely outperforming NAD^+^ IV (p < 0.01) and oral NR (p < 0.01) at the 3-h timepoint. The current study attempted to measure whole blood NAD^+^ concentrations at baseline and post infusion utilizing DBS protocols; however the assays were unable to detect changes due to insufficient sampling (Supplementary 2). This highlights the need for stable and standardized measurement of NAD^+^ concentrations in human blood, as has been called for in the literature (Balashova et al., 2022).

Oral NAD^+^ precursor supplementation has been demonstrated safe and effective in raising NAD^+^ levels without causing oxidative stress or liver enzyme elevations in healthy middle-aged adults (Martens et al., 2018; Sohouli et al., 2024), however limited data exists that examines oral NAD^+^ supplementation, NAD^+^ IV or NR IV applications with regard to changes in inflammation and liver enzymes. No studies have been reported to examine liver enzyme activity with NAD^+^ oral supplementation. One recent study did examine NAD^+^ IV application and noted a significant increase in bilirubin, a significant decrease in gamma glutamyl transferase (GGT), lactate dehydrogenase (LD), and AST with no change reported in ALP or ALT (Grant et al., 2019) at 8 h post-infusion. A second preprint study examined liver enzyme activity after one dose of NAD^+^ IV or NR IV and did not note significant changes at three or 24 h post-infusion (Hawkins et al., 2024). In the current study, there were significant decreases in ALP in the NAD^+^ IV group at 30-day post infusion, with no change in NR IV. No other changes in liver, renal or inflammation markers were noted in either group. Future investigations should also further examine eGFR, which was unable to be analyzed in the current study. Overall, no clinical significance was noted as all mean biomarkers were within normal ranges at baseline and 30-day post infusion and did not appear to affect hepatotoxicity (Table 3).

### 4.3 Metabolic outcomes

Exploratory metabolic outcomes in the current study were variable and should be interpreted with caution, due to the retrospective design, small sample size, absence of placebo or matched controls, and lack of standardized lifestyle controls. Further, longer duration study designs are required to examine markers like HbA1c, which is typically measured after 3 months. However, prior reports of metabolic alterations following oral NAD^+^ precursor supplementation support the need for further investigation. Two recent meta-analyses of controlled trials demonstrated NAD^+^ precursor oral supplementation had mixed effects on metabolic outcomes in humans (Zhong et al., 2022; Sohouli et al., 2024). Overall, supplementation with NAD^+^ precursors improved triglycerides, total cholesterol, LDLc and HDLc but resulted in hyperglycemia, compared with placebo or no treatment (Zhong et al., 2022). However, subgroup analysis demonstrated no statistically significant difference in any metabolic biomarkers after NR supplementation specifically (Zhong et al., 2022) although nicotinic acid and nicotinamide did increase fasting glucose and HbA1c compared to control (Sohouli et al., 2024). However, there are no data reported currently in oral NAD^+^ supplementation, NAD^+^ IV or NR IV. In the current study, the NR IV group, but not NAD^+^ IV group, demonstrated a significant reduction in HbA1c levels after 30 days with no changes noted in fasting glucose in either group. The relationship between NAD^+^ precursor supplementation and markers of glucose metabolism has been outlined previously, with NAD^+^ likely playing a multi-organ role in homeostasis (Yamaguchi and June 2017). However, conflicting results of the current study and the results of prior meta-analyses (Zhong et al., 2022; Sohouli et al., 2024) demonstrate the need for further study of individual precursors and outcomes.

Individual studies examining oral NR supplementation (Airhart et al., 2017), as well as a recent meta-analysis on various NAD^+^ precursors (Zhong et al., 2022), have demonstrated little to no effect on lipid metabolism in humans, particularly in relation to LDL cholesterol, total cholesterol, and triglycerides. In contrast, the current study found a significant reduction in HDL cholesterol levels and a non-significant increase in LDL cholesterol in the NAD^+^ group after 30 days. While the clinical relevance of these findings remains unclear, they may reflect shifts in lipid transport or reverse cholesterol efflux, potentially mediated by NAD^+^-dependent pathways such as sirtuin activity or hepatic lipid regulation (Dall et al., 2022). Further mechanistic studies are warranted to explore these unexpected outcomes.

Interestingly, in the current study, although diet and physical activity were not controlled for, changes in metabolic biomarkers occurred without significant differences in mean diet scores, physical activity, or body composition metrics from baseline to 30-day post-treatment. These findings suggest a possible association between NAD^+^ precursor administration and metabolic outcomes; however, the results are exploratory and require further evaluation in appropriately powered, controlled study designs.

This study had several notable strengths and limitations. Most notably, it is the first to directly compare intravenous NAD^+^ and NR products, both of which are already in widespread use across the commercial wellness market. In addition, this study specifically examined safety parameters, client experience, and preliminary data on related metabolic biomarkers. These data have not yet been reported in the literature. This study also has several limitations, including its retrospective design, small cohort size, and dosing constraints (i.e., 4-day “loading dose”) due to the commercial nature of the infusions. Notably, the absence of a placebo-controlled arm limits the generalizability and interpretation of the metabolic biomarker data. Finally, this study was designed and executed by a commercial entity that employed the researchers which may have resulted in implicit bias. Although, the study was exploratory and retrospective in nature, which does not imply causal inference. Nonetheless, the findings establish an important baseline for future, adequately powered clinical trials, a critical step given the increasingly prevalent use in the commercial market and the potential for future clinical applications. Future research studies need to explore both statistical significance and clinical relevance of findings.

### 4.4 Conclusion

In summary, this study provides a direct comparison of NAD^+^ IV and NR IV in a real world setting, offering important preliminary insights into safety, tolerability and metabolic impact. NR infusions were delivered more rapidly, with minor adverse experiences compared to NAD^+^. Both compounds appeared safe, with no clinically significant changes in liver enzymes and inflammation observed during and 30-day after infusion. Exploratory metabolic outcomes were variable and warrant further investigation. Additional studies are needed to assess long-term effectiveness beyond 30 days. Overall, these prelimary findings underscore the need for longer term, appropriately powered randomized placebo-controlled trials to determine effectiveness of NAD^+^ IV and its precursors.

## Data Availability

The data analyzed in this study is subject to the following licenses/restrictions: The data are not publicly available due to privacy and ethical restrictions. Requests to access these datasets should be directed to kirsten.reyna@uconn.edu.

## References

[B1] Adriouch S. Friedrich H. Boyer O. Seman M. Koch-Nolte F. (2012). Extracellular NAD(+): a danger signal hindering regulatory T cells. Microbes Infect. 14 (14), 1284–1292. 10.1016/j.micinf.2012.05.011 22634347

[B2] Ahmadi A. Begue G. Valencia A. P. Norman J. E. Lidgard B. Bennett B. J. (2023). Randomized crossover clinical trial of coenzyme Q10 and nicotinamide riboside in chronic kidney disease. JCI Insight 8 (11), e167274. 10.1172/jci.insight.167274 37159264 PMC10393227

[B3] Airhart S. E. Shireman L. M. Risler L. J. Anderson G. D. Nagana Gowda G. A. Raftery D. (2017). An open-label, non-randomized study of the pharmacokinetics of the nutritional supplement nicotinamide riboside (NR) and its effects on blood NAD+ levels in healthy volunteers. PloS One 12 (12), e0186459. 10.1371/journal.pone.0186459 29211728 PMC5718430

[B4] Amjad S. Nisar S. Bhat A. A. Shah A. R. Frenneaux M. P. Fakhro K. (2021). Role of NAD+ in regulating cellular and metabolic signaling pathways. Mol. Metab. 49 (July), 101195. 10.1016/j.molmet.2021.101195 33609766 PMC7973386

[B5] Balashova N. V. Zavileyskiy L. G. Artiukhov A. V. Shaposhnikov L. A. Sidorova O. P. Tishkov V. I. (2022). Efficient assay and marker significance of NAD+ in human blood. Front. Med. 9, 886485. 10.3389/fmed.2022.886485 35665345 PMC9162244

[B6] Berven H. Simon K. Sheard E. Søgnen M. Af Geijerstam S. A. Haugarvoll K. (2023). NR-SAFE: a randomized, double-blind safety trial of high dose nicotinamide riboside in parkinson’s disease. Nat. Commun. 14 (1), 7793. 10.1038/s41467-023-43514-6 38016950 PMC10684646

[B7] Campbell J. M. (2022). Supplementation with NAD+ and its precursors to prevent cognitive decline across disease contexts. Nutrients 14 (15), 3231. 10.3390/nu14153231 35956406 PMC9370773

[B8] Canto C. (2022). NAD+ precursors: a questionable redundancy. Metabolites 12 (7), 630. 10.3390/metabo12070630 35888754 PMC9316858

[B9] Chini C. C. S. Soares Cordeiro H. Tran N.Le K. Chini E. N. (2024). NAD metabolism: role in senescence regulation and aging. Aging Cell 23 (1), e13920. 10.1111/acel.13920 37424179 PMC10776128

[B10] Conze D. Brenner C. Kruger C. L. (2019). Safety and metabolism of long-term administration of NIAGEN (nicotinamide riboside chloride) in a randomized, double-blind, placebo-controlled clinical trial of healthy overweight adults. Sci. Rep. 9 (1), 9772. 10.1038/s41598-019-46120-z 31278280 PMC6611812

[B11] Covarrubias A. J. Perrone R. Grozio A. Verdin E. (2021). NAD+ metabolism and its roles in cellular processes during ageing. Nat. Rev. Mol. Cell Biol. 22 (2), 119–141. 10.1038/s41580-020-00313-x 33353981 PMC7963035

[B12] Dall M. Hassing A. S. Treebak J. T. (2022). NAD+ and NAFLD - Caution, causality and careful optimism. J. Physiology 600 (5), 1135–1154. 10.1113/JP280908 33932956

[B13] Damgaard M. V. Nielsen T. S. Basse A. L. Chubanava S. Trost K. Moritz T. (2022). Intravenous nicotinamide riboside elevates mouse skeletal muscle NAD+ without impacting respiratory capacity or insulin sensitivity. iScience 25 (2), 103863. 10.1016/j.isci.2022.103863 35198907 PMC8844641

[B14] Elhassan Y. S. Kluckova K. Fletcher R. S. Schmidt M. S. Garten A. Doig C. L. (2019). Nicotinamide riboside augments the aged human skeletal muscle NAD+ metabolome and induces transcriptomic and anti-inflammatory signatures. Cell Rep. 28 (7), 1717–1728.e6. 10.1016/j.celrep.2019.07.043 31412242 PMC6702140

[B15] Freeberg K. A. Udovich C. A. C. Martens C. R. Seals D. R. Craighead D. H. (2023). Dietary supplementation with NAD+-boosting compounds in humans: current knowledge and future directions. Journals Gerontology. Ser. A, Biol. Sci. Med. Sci. 78 (12), 2435–2448. 10.1093/gerona/glad106 37068054 PMC10692436

[B16] Gadegbeku C. A. Dhandayuthapani A. Zakaria Shrayyef M. Egan B. M. (2003). Hemodynamic effects of nicotinic acid infusion in normotensive and hypertensive subjects. Am. J. Hypertens. 16 (1), 67–71. 10.1016/s0895-7061(02)03196-5 12517686

[B17] Gasparrini M. Sorci L. Raffaelli N. (2021). Enzymology of extracellular NAD metabolism. Cell. Mol. Life Sci. CMLS 78 (7), 3317–3331. 10.1007/s00018-020-03742-1 33755743 PMC8038981

[B18] Grant R. Berg J. Mestayer R. Braidy N. Bennett J. Broom S. (2019). A pilot study investigating changes in the human plasma and urine NAD+ metabolome during a 6 hour intravenous infusion of NAD. Front. Aging Neurosci. 11, 257. 10.3389/fnagi.2019.00257 31572171 PMC6751327

[B19] Gross C. J. Henderson L. M. (1983). Digestion and absorption of NAD by the small intestine of the rat. J. Nutr. 113 (2), 412–420. 10.1093/jn/113.2.412 6218262

[B20] Grozio A. Mills K. F. Yoshino J. Bruzzone S. Sociali G. Tokizane K. (2019). Slc12a8 is a nicotinamide mononucleotide transporter. Nat. Metab. 1 (1), 47–57. 10.1038/s42255-018-0009-4 31131364 PMC6530925

[B21] Harries A. D. Heatley R. V. (1983). Nutritional disturbances in crohn’s disease. Postgrad. Med. J. 59 (697), 690–697. 10.1136/pgmj.59.697.690 6359105 PMC2417669

[B22] Hawkins J. Idoine R. Kwon J. Shao A. Dunne E. Hawkins E. (2024). Randomized, placebo-controlled, pilot clinical study evaluating acute niagen®+ IV and NAD+ IV in healthy adults. Prepr. medRxiv. 10.1101/2024.06.06.24308565

[B23] Hays R. D. Bjorner J. B. Revicki D. A. Spritzer K. L. Cella D. (2009). Development of physical and mental health summary scores from the patient-reported outcomes measurement information system (PROMIS) global items. Qual. Life Res. An Int. J. Qual. Life Aspects Treat. Care Rehabilitation 18 (7), 873–880. 10.1007/s11136-009-9496-9 19543809 PMC2724630

[B24] Kane A. E. Sinclair D. A. (2018). Sirtuins and NAD+ in the development and treatment of metabolic and cardiovascular diseases. Circulation Res. 123 (7), 868–885. 10.1161/CIRCRESAHA.118.312498 30355082 PMC6206880

[B55] Kassambara A. (2023). rstatix: Pipe-Friendly Framework for Basic Statistical Tests. R package version 0.7.2. Available online at: https://rpkgs.datanovia.com/rstatix/ .

[B25] Kleta R. Romeo E. Ristic Z. Ohura T. Stuart C. Arcos-Burgos M. (2004). Mutations in SLC6A19, encoding B0AT1, cause hartnup disorder. Nat. Genet. 36 (9), 999–1002. 10.1038/ng1405 15286787

[B26] Kropotov A. Kulikova V. Nerinovski K. Yakimov A. Svetlova M. Solovjeva L. (2021). Equilibrative nucleoside transporters mediate the import of nicotinamide riboside and nicotinic acid riboside into human cells. Int. J. Mol. Sci. 22 (3), 1391. 10.3390/ijms22031391 33573263 PMC7866510

[B27] Lai J.-S. Cella D. Choi S. Junghaenel D. U. Christodoulou C. Gershon R. (2011). How item banks and their application can influence measurement practice in rehabilitation medicine: a PROMIS fatigue item bank example. Archives Phys. Med. Rehabilitation 92 (10), S20–S27. 10.1016/j.apmr.2010.08.033 21958919 PMC3696589

[B28] Lai J.-S. Wagner L. I. Jacobsen P. B. Cella D. (2014). Self-reported cognitive concerns and abilities: two sides of one coin? Psycho-Oncology 23 (10), 1133–1141. 10.1002/pon.3522 24700645 PMC4185008

[B29] Lautrup S. Sinclair D. A. Mattson M. P. Fang E. F. (2019). NAD+ in brain aging and neurodegenerative disorders. Cell Metab. 30 (4), 630–655. 10.1016/j.cmet.2019.09.001 31577933 PMC6787556

[B30] Lee P. H. Macfarlane D. J. Lam T. H. Stewart S. M. (2011). Validity of the international physical activity questionnaire short form (IPAQ-SF): a systematic review. Int. J. Behav. Nutr. Phys. Activity 8 (1), 115. 10.1186/1479-5868-8-115 22018588 PMC3214824

[B31] Lyo (2026). “NAD+ injection (Lyo),” in Empower pharmacy. Available online at: https://www.empowerpharmacy.com/compounding-pharmacy/nad-injection/(Accessed January 3, 2026).

[B32] Martens C. R. Denman B. A. Mazzo M. R. Armstrong M. L. Reisdorph N. McQueen M. B. (2018). Chronic nicotinamide riboside supplementation is well-tolerated and elevates NAD+ in healthy middle-aged and older adults. Nat. Commun. 9 (1), 1286. 10.1038/s41467-018-03421-7 29599478 PMC5876407

[B33] McLester C. N. Nickerson B. S. Kliszczewicz B. M. McLester J. R. (2020). Reliability and agreement of various InBody body composition analyzers as compared to dual-energy X-Ray absorptiometry in healthy men and women. J. Clin. Densitom. Official J. Int. Soc. Clin. Densitom. 23 (3), 443–450. 10.1016/j.jocd.2018.10.008 30472111

[B34] Nelson R. H. Vlazny D. Smailovic A. Miles J. M. (2012). Intravenous niacin acutely improves the efficiency of dietary fat storage in lean and Obese humans. Diabetes 61 (12), 3172–3175. 10.2337/db12-0236 22923472 PMC3501872

[B35] Niacin (2025). Office of dietary supplements - niacin. Available online at: https://ods.od.nih.gov/factsheets/Niacin-HealthProfessional/(Accessed May 26, 2025).

[B36] Nikiforov A. Dölle C. Niere M. Ziegler M. (2011). Pathways and subcellular compartmentation of NAD biosynthesis in human cells: from entry of extracellular precursors to mitochondrial NAD generation. J. Biol. Chem. 286 (24), 21767–21778. 10.1074/jbc.M110.213298 21504897 PMC3122232

[B37] O’hollaren P. (1961). Diphosphopyridine nucleotide in the prevention, diagnosis and treatment of drug addiction. A preliminary report. West. J. Surg. Obstetrics, Gynecol. 69, 213–215. 13730082

[B38] PROMIS Pain Intensity (2025). “Instrument: PROMIS pain intensity - short form 3a v1.0,” in NIDA CTN common data elements. Available online at: https://cde.nida.nih.gov/instrument/0a481bfb-a5e6-3c84-e050-bb89ad43314d (Accessed February 19, 2025).

[B40] Ratajczak J. Joffraud M. Trammell S. A. J. Ras R. Canela N. Boutant M. (2016). NRK1 controls nicotinamide mononucleotide and nicotinamide riboside metabolism in mammalian cells. Nat. Commun. 7 (October), 13103. 10.1038/ncomms13103 27725675 PMC5476803

[B41] Rifas-Shiman S. L. Willett W. C. Lobb R. Kotch J. Dart C. Gillman M. W. (2001). PrimeScreen, a brief dietary screening tool: reproducibility and comparability with both a longer food frequency questionnaire and biomarkers. *Public Health Nutr.* Engl. 4 (2), 249–254. 10.1079/phn200061 11299098

[B43] Saqr A.-H. A. Kamali C. Brunnbauer P. Haep N. Koch P. Hillebrandt K. H. (2023). Optimized protocol for quantification of extracellular nicotinamide adenine dinucleotide: evaluating clinical parameters and pre-analytical factors for translational research. Front. Med. 10, 1278641. 10.3389/fmed.2023.1278641 38259852 PMC10800990

[B44] Sauve A. A. Wang Q. Zhang N. Kang S. Rathmann A. Yang Y. (2023). Triple-isotope tracing for pathway discernment of NMN-induced NAD+ biosynthesis in whole mice. Int. J. Mol. Sci. 24 (13), 11114. 10.3390/ijms241311114 37446292 PMC10342116

[B45] Sohouli M. H. Tavakoli S. Reis M. G. Hekmatdoost A. Guimarães N. S. (2024). Changes in glucose metabolism, C-Reactive protein, and liver enzymes following intake of NAD + precursor supplementation: a systematic review and meta‐regression analysis. Nutr. and Metabolism 21 (1), 35. 10.1186/s12986-024-00812-0 38915015 PMC11195006

[B46] Trammell S. A. J. Schmidt M. S. Weidemann B. J. Redpath P. Jaksch F. Dellinger R. W. (2016). Nicotinamide riboside is uniquely and orally bioavailable in mice and humans. Nat. Commun. 7 (October), 12948. 10.1038/ncomms12948 27721479 PMC5062546

[B47] Weinfurt K. P. Lin Li Bruner D. W. Cyranowski J. M. Dombeck C. B. Hahn E. A. (2015). Development and initial validation of the PROMIS(®) sexual function and satisfaction measures version 2.0. J. Sex. Med. 12 (9), 1961–1974. 10.1111/jsm.12966 26346418

[B56] Wickham H. Averick M. Bryan J. Chang W. McGowan L. D. François R. (2019). Welcome to the tidyverse. J. Open Source Softw. 4 (43), 1686. 10.21105/joss.01686

[B48] Wu J. Jin Z. Zheng H. Yan L. J. (2016). Sources and implications of NADH/NAD(+) redox imbalance in diabetes and its complications. Diabetes, Metabolic Syndrome Obes. Targets Ther. 9, 145–153. 10.2147/DMSO.S106087 27274295 PMC4869616

[B49] Wu C.-Yi Kupferschmid A. C. Chen L. McManus A. J. Kivisäkk P. Galler J. A. (2025). Cognitive and alzheimer’s disease biomarker effects of oral nicotinamide riboside (NR) supplementation in older adults with subjective cognitive decline and mild cognitive impairment. Alzheimer’s and Dementia (New York, N. Y.) 11 (1), e70023. 10.1002/trc2.70023 39817194 PMC11733434

[B50] Yamaguchi S. Jun Y. (2017). Adipose tissue NAD+ biology in obesity and insulin resistance: from mechanism to therapy. BioEssays News Rev. Mol. Cell. Dev. Biol. 39 (5), 1600227. 10.1002/bies.201600227 PMC5469033 28295415

[B51] Yogi T. N. Pooja B. C. Bhusal A. Limbu S. Kafle R. (2024). Alcoholic pellagrous encephalopathy: a case report on atypical presentation and diagnostic dilemma in alcohol-related disorders. Ann. Med. Surg. 86 (1), 501–506. 10.1097/MS9.0000000000001497 38222748 PMC10783386

[B52] Yu L. Buysse D. J. Germain A. Moul D. E. Stover A. Dodds N. E. (2011). Development of short forms from the PROMIS^TM^ sleep disturbance and sleep-related impairment item banks. Behav. Sleep. Med. 10 (1), 6–24. 10.1080/15402002.2012.636266 22250775 PMC3261577

[B53] Zhang J. Wang H.-L. Lautrup S. Nilsen H. L. Treebak J. T. Watne L. O. (2025). Emerging strategies, applications and challenges of targeting NAD+ in the clinic. Nat. Aging 5 (9), 1704–1731. 10.1038/s43587-025-00947-6 40926126

[B54] Zhong Ou Wang J. Tan Y. Lei X. Tang Z. (2022). Effects of NAD+ precursor supplementation on glucose and lipid metabolism in humans: a meta-analysis. Nutr. and Metabolism 19 (1), 20. 10.1186/s12986-022-00653-9 35303905 PMC8932245

