# Supplementary material for: Intravenous infusion of nicotinamide adenine dinucleotide (NAD+) versus nicotinamide riboside (NR): a retrospective tolerability pilot study in a real-world setting
Source: Front Aging. 2026 Feb 2;7:1652582. doi: 10.3389/fragi.2026.1652582 (PMC12907335; doi:10.3389/fragi.2026.1652582)
Supplement: Supplementary file 1 [file Supplementaryfile1.docx]

Supplementary Material 1

Table 1A The means and standard deviations for NAD^+^ clients at baseline and 30-days post-infusion

**Table 1A – NAD^+^ Averages at Baseline and 30-day Post-infusion**

| Biomarker | Average at baseline | Average 30-days post-infusion |
| --- | --- | --- |
| ALP | 78.33 (28.43) | 77.4 (20.26) |
| ALT | 31.67 (34.93) | 36.2 (35.83) |
| AST | 29.33 (18.77) | 34.2 (23.85) |
| BUN | 11.67 (1.53) | 14.6 (5.68) |
| Creatinine | 0.86 (0.03) | 0.99 (0.27) |
| Glucose (Fasting) | 89.33 (6.11) | 92.8 (18.27) |
| HDLc | 65.6 (21.66) | 59.8 (18.77) |
| HbA1c | 5.36 (0.22) | 5.38 (0.22) |
| LDLc | 107.4 (28.18) | 117.2 (35.85) |
| TSH | 1.74 (0.87) | 1.8 (1.75) |
| Triglycerides | 120.2 (68.26) | 103 (62.67) |
| hsCRP | 1.38 (1.33) | 2 (1.81) |

Table 1B The means and standard deviations for NR clients at baseline and 30-days post-infusion

**Table 1B - NR Averages at Baseline and 30-day Post-infusion**

| Biomarker | Average at baseline | Average 30days post-infusion |
| --- | --- | --- |
| ALP | 59.14 (16.7) | 63.88 (15.99) |
| ALT | 17.86 (7.17) | 19 (7.93) |
| AST | 19.86 (3.58) | 18.63 (5.07) |
| BUN | 13.14 (2.27) | 15 (3.25) |
| Creatinine | 0.92 (0.25) | 0.95 (0.19) |
| Glucose (Fasting) | 85.86 (5.11) | 89.25 (12.81) |
| HDLc | 61.63 (14.05) | 61.75 (12.16) |
| HbA1c | 5.41 (0.31) | 5.29 (0.29) |
| LDLc | 125.13 (26.34) | 126 (29.56) |
| TSH | 2.07 (0.89) | 2.09 (1.21) |
| Triglycerides | 96.75 (36.19) | 87 (24.46) |
| hsCRP | 1.46 (0.85) | 1.99 (2.79) |
